# *In Situ* Wood Fiber Dyeing Through Laccase Catalysis for Fiberboard Production

**DOI:** 10.3389/fbioe.2021.778971

**Published:** 2021-12-03

**Authors:** Alberto Colella, Addolorata De Chiaro, Vincenzo Lettera

**Affiliations:** ^1^ Biopox srl, Viale Maria Bakunin, Napoli, Italy; ^2^ Department of Chemical Science, University of Naples Federico II, Napoli, Italy

**Keywords:** *in situ* dyeing, medium-density fiberboard, POXA1b, enzyme oxidation, colors

## Abstract

The aim of the present work was to develop an innovative and environmentally friendly process for wood fiber dyeing and to produce 3-dimensionally fully colored medium-density fiberboard (MDF). The potential of laccase-catalyzed polymerization of selected precursors to form dyes useful in fiberboard manufacturing, a technique used for the first time in this field, was demonstrated. Some of the 7 aromatic compounds tested yielded colored products after laccase treatment under both acid and alkaline conditions, and a good variety of colors was attained by using mixtures of two different monomers. To demonstrate the coloration and design potential of laccase conversion of aromatic compounds, MDFs were enzymatically dyed using an *in situ* one-step laccase-catalyzed coloration process, and the results were compared against commercial MDFs obtained by using organic coloring agents. Important advantages over conventional processing methods include good color fastness and, in some cases, new hydrophobic properties, allowing designers and woodworkers to explore the beauty of textures and the use of simpler and milder processing conditions that eliminate harsh chemical use and reduce energy consumption.

## Introduction

In the frame of circular economy, wood wastes are becoming increasingly attractive to meet the enormous demand of wood that exists in a wide range of industries (Euring et al., 2011). When trees are processed, wood chips and sawdust are produced, in addition to thinning and residual wood. These residues can be used as raw material for furniture and interior design in the production of wood-based devices, such as wood medium-density fiberboard (MDF). Wood fibers are generally blended with resin, wax, and other additives; dried; and pressed. After pressing, the boards are cooled, sanded, trimmed, and sawed to final dimensions. MDFs may also be painted or laminated; however, the coloring procedure generally covers only the small part of wood fibers visible on the surface.

At the end of the last century, Valbopan S.A. (Leiria, Portugal) presented a special wood-based fiberboard with a “wooden natural” surface and with color throughout the thickness to the market. Manufacturers, architects, and technicians have demonstrated technical, aesthetic benefits, and advantages of its use. The final product is a panel made of wood fibers colored during the production process. Although this technology results in an innovation in the dyeing process, that is, third dimensional dyeing, the fibers still need to be soaked with organic colorants and bounded to one another through chemical agents, which usually result to be toxic or recalcitrant.

The potential for the use of wood wastes as ecofriendly, sustainable, recyclable, and reusable product is not fully exploited yet because this requires new processes to be set up along the entire value-added chain, from the adhesive used during fabrication to the chemical components used in the dyeing/laminating process. As a fact, the trend in the global market has shifted toward the use of fiberboards not containing toxic, hazardous, or oil-derived chemicals (Euring et al., 2011). In this perspective, the forest industry is increasingly adopting enzyme-based technologies for binderless or self-bonding fiberboard manufacturing (Vitronea et al., 2021).

Wood-decay or parasitic fungi are generally able to digest wood; thus, their enzymes have stimulated several studies on the exploitability of fungal enzymes in the forest industry (López et al., 2017; Andlar et al., 2018; Zhuo and Fan, 2021). Among them, laccases (benzenediol: oxygen oxidoreductase; EC 1.10.3.2) have been extensively characterized because of their potential for industrial applications. These enzymes, belonging to the multicopper oxidase family, catalyze the monoelectronic oxidation of a broad range of substrates, such as phenols and aromatic or aliphatic amines, to their corresponding radicals, using molecular oxygen. Laccases can oxidize aromatic polymers, such as lignin, through free radical reactions (Sun et al., 2013). This catalytic activity has been applied to activate lignin to produce lignin-based adhesives and to attain physically and/or chemically modified wood fibers (Rowell, 1996; Hu et al., 2016; Agustin et al., 2021).

Most of the laccase-based approaches, related to wood fiber treatment, have been focused on the improvement of MDF mechanical properties through the augmentation of bonding strength within and between the fibers (Nasir et al., 2014; Nasir et al., 2016). Surprisingly, there is a complete lack of data concerning laccase applications in the wood dyeing sector. As a fact, laccases are able to catalyze coupling of aromatic compounds, such as phenols and diamines, producing colored oligomers or polymers in the presence of different solid porous matrixes (Su et al., 2018). Several research groups have already applied *in situ* enzymatic synthesis of dyes in existing industrial products such as cotton, wool, and hair dye (Kim et al., 2007; Jeon et al., 2010; Pezzella et al., 2016; Sousa et al., 2016; Kumar et al., 2018; Prajapati et al., 2018; Yuan et al., 2018).

In our opinion, eco-compatibility and catalyzing properties of laccases could be applied to the *in situ* dyeing of wood fibers. MDF dyeing works by penetrating the wood fibers, soaking in and drying in the wood pores. In order to successfully and permanently dye MDFs, dyes have to be applied before the panel is sealed by polyurethane or other finishing agents.

The aim of this research was to develop a laccase-catalyzed *in situ* dyeing process for wood fibers that could be integrated into the already existing industrial processes, as an alternative to conventional MDF dyeing methods. Several studies, exploring laccase catalysis, suggest that a wide range of color diversity may be achieved through a combination of different precursors incubated together in the presence of enzymes (Kim et al., 2007; Jeon et al., 2010; Pezzella et al., 2016; Kumar et al., 2018; Prajapati et al., 2018; Yuan et al., 2018). This study was focused on investigating the dyeing reactions using laccase catalysis on seven selected substrates combined in heteromolecular mixtures that led to formation of mixtures of heteropolymers or homopolymers. Color parameters of heteromolecular mixture reactions were compared with those of single monomer reactions, by also considering reaction processing parameters: acid and alkaline pH and reaction times. Furthermore, wood fiber wettability was tested to determine the related functionalization degree, due to the modification of their chemical surface properties. To date, this is the first reported application of laccases in wood dyeing.

## Materials and Methods

### Materials

Recombinant POXA1b laccase from *Pleurotus ostreatus*, expressed in the yeast *Pichia pastoris* under the control of the AOX1 promoter, was produced by BioPox srl. Bleached pine wood fibers were provided by Wetlands Engineering (Belgium). The wood fibers were stored at 22 ± 3°C in a hermetically sealed plastic bag under dry conditions.

Valchromat^®^ commercial fiberboard samples were provided by a local joinery. As reported in the related datasheet, the fiberboards were colored during the production process with organic coloring agents and chemically bonded by a melamine–urea–formaldehyde resin. All reagents were purchased from Sigma-Aldrich Corp. (St. Louis, MO).

### Enzyme Activity Measurement

Laccase activity was determined using 2,2′-azino-bis (3-ethylbenzothiazoline-6-sulfonate) (ABTS) as a substrate following the reaction at 420 nm (ε_420_ = 36 × 10^3^ M^−1^ cm^−1^), as previously reported (Lettera et al., 2010).

### Dye Synthesis

The following chemicals were used as precursors for the dye synthesis: resorcinol, *p*-phenylenediamine, *m*-aminophenol, 4,5-diamino-1-(2-hydroxyethyl)pyrazole sulfate, 2,4,5,6-tetraaminopyrimidine sulfate, syringic acid, and 2,5-diaminobenzenesulfonic acid (2,5-DABSA). All precursors were purchased from Sigma-Aldrich Corp. (St. Louis, MO).

The reactions were carried out through incubation of 5 mM of each precursor dissolved in 100 mM sodium citrate at pH 3 or in 50 mM Tris–HCl at pH 9. Heteromolecular synthesis was achieved by mixing a couple of precursors in a 1:1 M ratio. Each solution was incubated with 1U/ml of laccase in aqueous solution up to 24 h at 22 ± 3°C without stirring. The reactions were followed by registering the absorbance spectra (400–800 nm) at the beginning of the reaction and after 1, 5, and 24 h and subtracting spectra of substrates in the absence of enzyme as blank (UV-1600PC VWR spectrophotometer). All measurements were performed in triplicate, and the standard deviation of the absorbances was evaluated at the λ_max_ for each assay.

### *In situ* Wood Fiber Dyeing

Wood fibers (1.5 g) were dyed by soaking the fiber bulk in 50 ml of dyeing solution. Each dyeing bath was formulated by 5 mM of each precursor in homogeneous solution or by 5 mM of each precursor in a heteromolecular mixture in both pH conditions and incubated with laccase. The laccase activity was increased up to 40U/ml, in order to allow homogeneous dyeing of the fibers and to enhance the low diffusivity of the catalyzer into the solid matrix. The dyeing procedure was carried out for 5 h.

Wood fibers treated with dyeing solutions, which contain precursors in the absence of enzymes or laccases in the absence of oxidizable precursors, were used as control. The treated wood fibers were washed thoroughly with deionized water until the washing solution became colorless. Finally, the wet fibers were dried at 40°C for 7 days in the oven.

### Form-Pressing Wood Fibers

1.5 g of wet dyed fibers were directly blown into a tube with a cross-sectional area of 9.6 cm^2^ and constantly pressed using a press (manual hydraulic press 10000 Kg-10 T with pressure gauge MA PE PE10, EchoENG, Italy) at 10 KPa for 5 days at 40°C to fabricate a mat of uniform thickness. Finally, a form-stable, form-pressed dried sample was removed from the press.

### Color Values

The colorimeter Mightex’s SLB-1200-1 universal LED driver was used to determine the color values L* (lightness to darkness), a*(redness to greenness), and b* (yellowness to blueness) of each enzymatically treated and untreated sample, represented by the CIELAB color space system. Each sample of undiluted colored solutions or pressed colored wood fibers was measured three times. An average color measurement was calculated from the data collected for each sample in triplicate. Color values were converted into the RGB system through the conversion tool available online (colorizer.org), in order to graphically represent the variety of colors achieved in liquid solutions. When the RGB colors related to the replicates of each experiment appeared indistinguishable to the human eye, the variability was considered negligible.

### Color Fastness to Water

Color fastness to water of the wood fibers dyed was evaluated by dipping dyed samples in 200 ml of hot water (80°C) for 2 h, as reported by Zhu and coworkers (Zhu et al., 2018). Afterward, the color values of the treated samples were recorded using a colorimeter. Color differences between two samples were represented as ΔE and were calculated using Eq. (1) (Schanda, 2007):

$$\Delta \text{E}={\left[\left(\Delta \text{L}*\right)2+\left(\Delta \text{a}*\right)2+\left(\Delta \text{b}*\right)2\right]}^{1/2},$$ (1)

where ΔL*, Δa*, and Δb* represent the differences between the corresponding units of each sample.

Surface color change values of wood fibers before and after dyeing were determined through solid color measurement using the CIELAB color system. The color values were measured in three portions of each sample. The average value of each chromaticity parameter was recorded, and the total color difference (ΔE) was calculated using Eq. (1).

The total color difference ΔE, obtained using Eq. (1), was used to measure the color fastness in response to treatment with water. All measurements of color values were performed in triplicate, and the corresponding standard deviations were evaluated as ΔE. A difference in colors, which corresponded to a ΔE less than 3, was indistinguishable to the human eye, and the related variability was considered negligible.

### Contact Angle and Drop Absorption Time Measurements

The water contact angle was measured with a deionized water droplet on the surface of the form-pressed dried sample at 22 ± 3°C. A dosing volume of the droplet was set at 20 µL using a Hamilton 500-µL syringe. The receiving surface was placed on the stationary drum, and a picture of the drop-receiving surface interaction was taken by using a transversely positioned digital camera (Sony 2.6 pol LCD with built-in flash 20.1 MP) in the auto mode. Measurements were taken 1 s after the drop release. The images were analyzed by ImageJ 1.45s Drop Shape Analysis plugin. Moreover, by measuring the time at which the drop touches the wood fiber surface and the time at which the drop disappears from the surface, we determined the drop absorption time of the sample. The data were calculated by averaging values after measuring at three different points on each sample.

## Results

### Analyses of Laccase-Catalyzed Dyes Toward Monomer Oxidation in Water Solutions at Acid and Alkaline pH

The ability of POXA1b laccase to catalyze dye formation when incubated with seven commercially available compounds was first assayed in aqueous solution in both acidic and alkaline environments.

**FIGURE 1:**
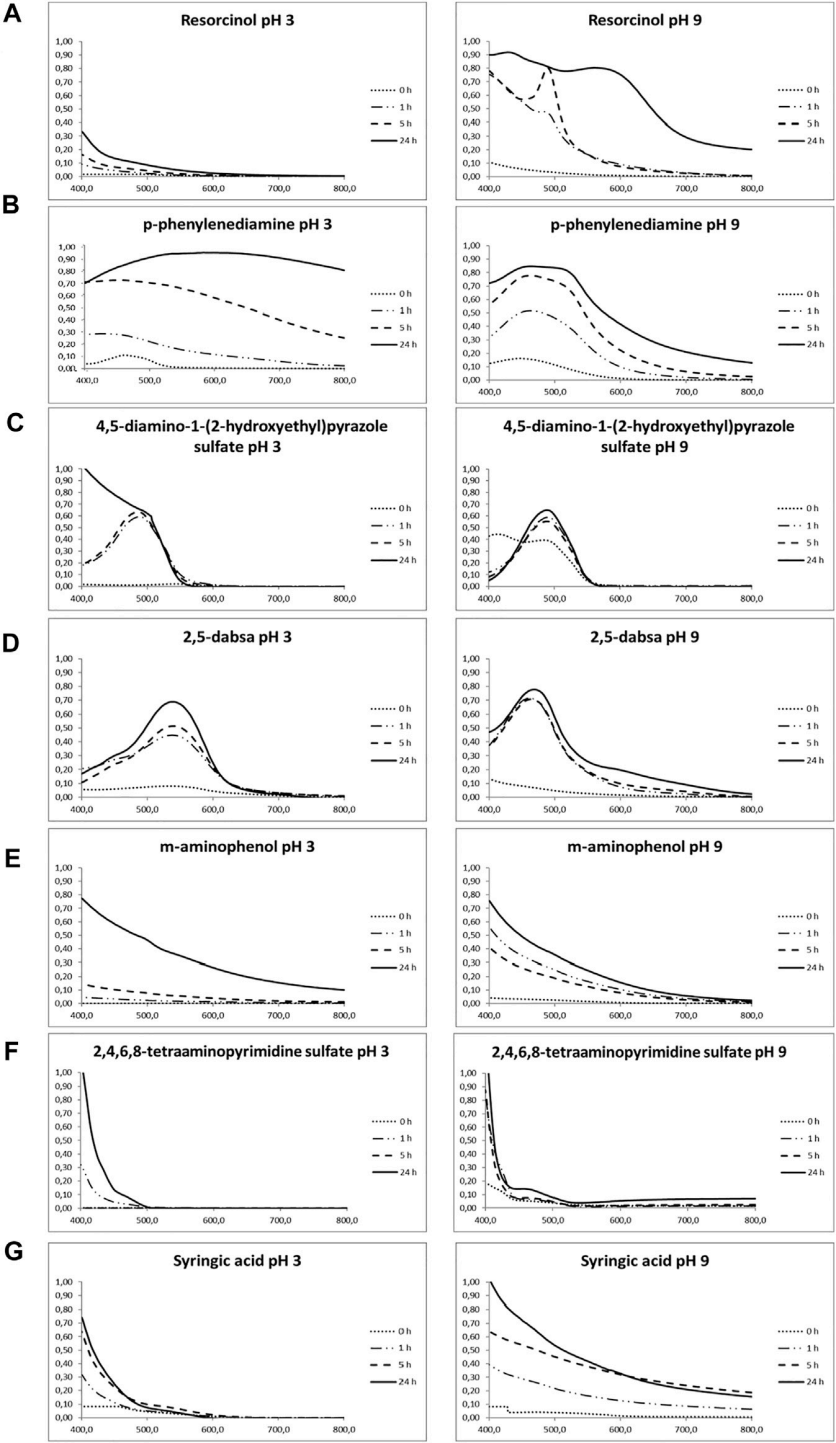
Time-dependent visible light absorption spectra of the reacted solutions obtained at different times during the oxidation experiments. Diluted samples incubated with laccase were indicated in bracket. **(A)** Resorcinol (5 h pH 9, 1:2 diluted; 24 h pH 9, 1:8 diluted). **(B)** *p*-phenylenediamine (1 h pH 3, 1:10 diluted; 5 h pH 3, 1:10 diluted; 24 h pH 3, 1:12 diluted) (1 h pH 9, 1:4 diluted; 5 h pH 9, 1:5 diluted; 24 h pH 9, 1:7 diluted). **(C)** 4,5-diamino-1-(2-hydroxyethyl)pyrazole sulfate (1 h pH 3, 1:2 diluted; 5 h pH 3, 1:4 diluted; 24 h pH 3, 1:15 diluted) (1 h pH 9, 1:20 diluted; 5 h pH 9, 1:80 diluted; 24 h pH 9, 1:100 diluted). **(D)** 2,5-diaminobenzenesulfonic acid (0 h pH 3, 1:10 diluted; 1 h pH 3, 1:20 diluted; 5 h pH 3, 1:40 diluted; 24 h pH 3, 1:40 diluted) (1 h pH 9, 1:4 diluted; 5 h pH 9, 1:10 diluted; 24 h pH 9, 1:20 diluted). **(E)** *m*-aminophenol (1 h pH 9, 1:2 diluted; 5 h pH 3, 1:4 diluted; 24 h pH 3, 1:10 diluted). **(F)** 2,4,5,6-tetraaminopyrimidine sulfate (1 h pH 9, 1:4 diluted; 5 h pH 3, 1:4 diluted; 24 h pH 3, 1:4 diluted). **(G)** syringic acid (1 h pH 3, 1:4 diluted; 5 h pH 3, 1:4 diluted; 24 h pH 3, 1:4 diluted) (1 h pH 9, 1:4 diluted; 5 h pH 9, 1:4 diluted; 24 h pH 9, 1:5 diluted). Standard deviation of absorbance at λmax was less than 8%. Related controls are reported in Supplementary Figure S1.

**FIGURE 2:**
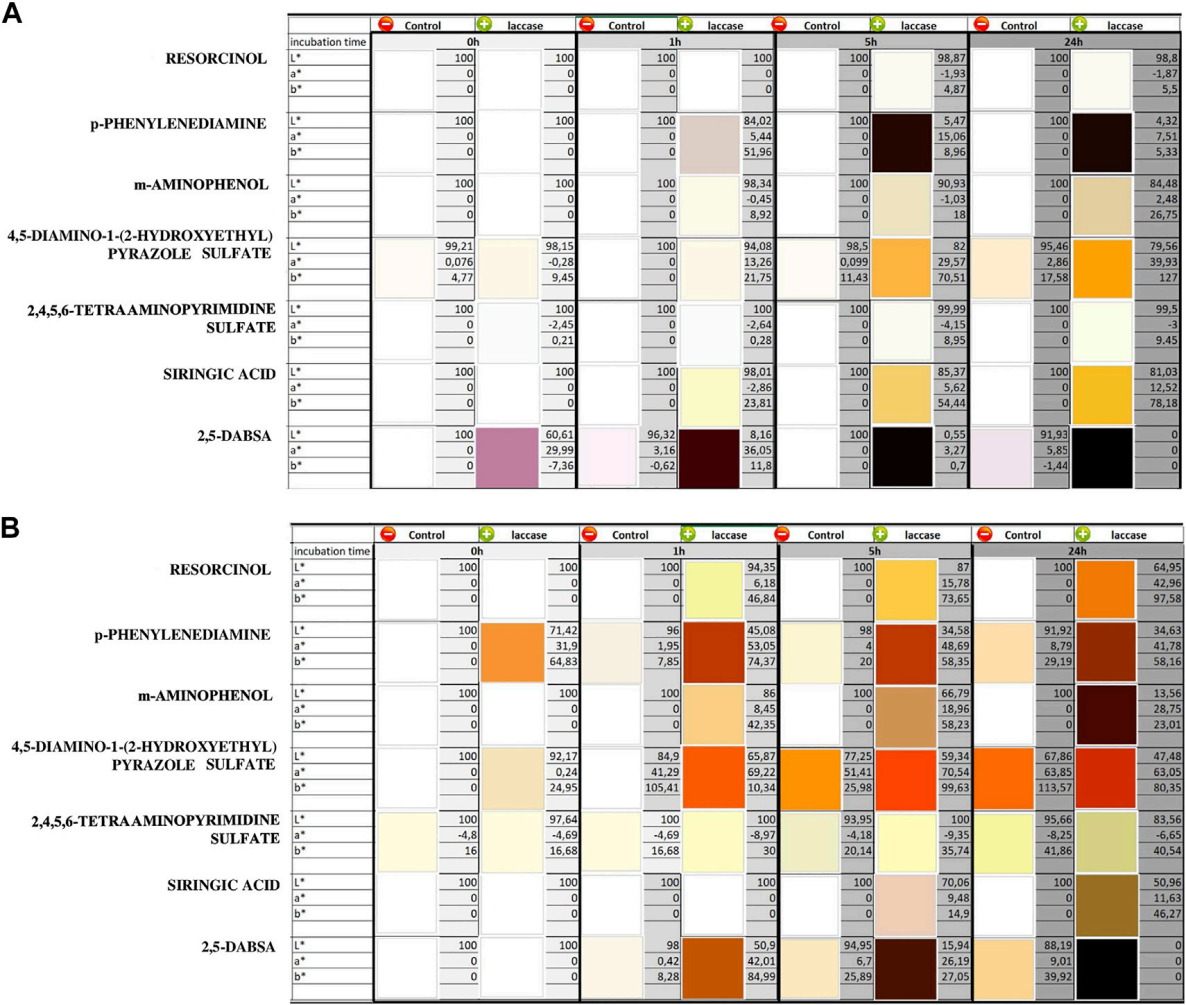
CIELAB color values and related color images of the reacted solutions containing one monomer at different times at pH 3 **(A)** and pH 9 **(B)**. The solutions were evaluated at different time of laccase incubation (0, 1, 5, and 24 h). The controls were analyzed under the same conditions by means of heat-inactivated laccase. The standard deviations were less than 3 units of ΔE for each color value measurement.

Dye generation catalyzed by laccase after different periods of incubation was observed, and the spectrum of each solution was monitored by visible spectrophotometry (Figure 1). Heat-inactivated laccase was used as a negative control to monitor the spontaneous oxidation reaction, which occurred in the absence of laccase activity in the dyeing solution (Supplementary Figure S1). Color generation through spontaneous and laccase-induced reactions was measured by means of colorimetric analysis, and the related CIELAB coordinates L*, a*, and b* were also reported (Figure 2).

For resorcinol (Figure 1A), the color of solution changed from colorless to orange during enzymatic oxidation at pH 9; however, no remarkable absorption band in the visible region was detected under acidic conditions. Conversely, an evident increase in absorption in the visible spectrum with a peak centered at around 490 nm was observed during the enzymatic incubation under alkaline conditions up to 5 h. After 24 h, the total absorption still increased, but this peak was not more detectable, and two others appeared centered at about 420 and 580 nm.

The visible spectra of *p*-phenylenediamine oxidation exhibited a remarkable increase in absorption intensity centered in the range from 400 to 470 nm, in both pH conditions, due to the electronic transition of quinoneimine structures in the polymer chains (Figure 1B). A red shift occurred after 24 h of incubation under acidic conditions, whereas a second peak at 500-550 nm appeared and increased during the incubation time under alkaline conditions. The color solution changed to dark brown under acidic conditions and red/light brown under alkaline conditions.

Spectrophotometric analysis of 4,5-diamino-1-(2-hydroxyethyl)pyrazole sulfate displayed a peak at about 490 nm, starting from the first hour of laccase action (Figure 1C). As the reaction continued, the absorbance remarkably increased, keeping the same curve shape for all the time points but 24 h at pH 3, when the absorbance tended to increase in the ultraviolet wavelength direction. The color variation of the solution in the acid environment was not marked well, and a dark brown hue was detectable at 24 h of enzyme incubation at pH 9.

POXA1b was also able to oxidize 2,5-diaminobenzenesulfonic acid under both acidic and alkaline conditions (Figure 1D). In fact, a remarkable increase in absorption intensity was measured, with peaks centered at around 540 and 460 nm at pH 3 and pH 9, respectively. Treatments at both pH produced colored products from cyclamen to dark violet/brown under the acidic condition and from orange to dark brown in the alkaline solution.

The laccase oxidation of *m*-aminophenol, 2,4,5,6-tetraaminopyrimidine sulfate, and syringic acid showed similar patterns: a rapid decrease in absorbance with the wavelength increasing in the visible spectrum (Figure 1 from E to G). From the absorption spectra presented in Figure 1E, we can clearly observe that the reactions, under both pH conditions, increased the light absorption ability and that the enhancement of light absorption increased with the increase in the reaction time. The light absorption was higher close to the ultraviolet range and decreased with increasing wavelength.

As far as *m*-aminophenol is concerned, a faint color variation was detectable at pH 3 only after 24 h of enzyme incubation, whereas brown color was produced after 5 h in the alkaline environment. A more pronounced yellow color was originated from the enzymatic solution containing syringic acid at pH 3 after 5 h, whereas dark beige was produced at pH 9.

Finally, laccase seems to be inactive on 2,4,5,6-tetraaminopyrimidine sulfate at both pH because no detectable difference occurred with respect to the controls. Considering the whole of reactions, it is not possible to rule out the existence of spontaneous oxidation in the absence of the enzyme, but for most of the compounds, a significant increase in the reaction rate is observable in the presence of laccase for both pH conditions. Most of the precursors gave colorless or slightly colored solutions when dissolved in water in the absence of oxidizers (Figure 2). On the other hand, addition of laccase resulted in visible color generation. Overall, the laccase-induced oxidation rate was higher in alkaline conditions than in the acidic ones, and the generated colors related to each compound at both pH significantly differed from each other in CIELAB coordinates.

### Increasing the Color Palette Through the Oxidation of Heteromolecular Mixture

The synthesis of new colors was achieved by mixing of two monomers in aqueous solution in a 1:1 M ratio. Analogously to the previous experiments, all the twenty-one combinations derived from the coupling of the seven precursors were incubated with laccase at both pH 3 and pH 9.

Since the color related to the monomer polymerization changes with polymerization extent, which, in turn, depends on the polymerization time, the CIELAB and visible spectra of each reaction were measured over time. The color contribution, due to the autoxidation tendency of the heteromolecular mixtures, was evaluated by incubating each solution with heat-inactivated laccase (Supplementary Table S2). The color parameters attained by the oxidation of the heteromolecular mixtures were also compared with the values related to the same blending solutions, whose monomers had been previously oxidized separately (Supplementary Table S3).

**FIGURE 3:**
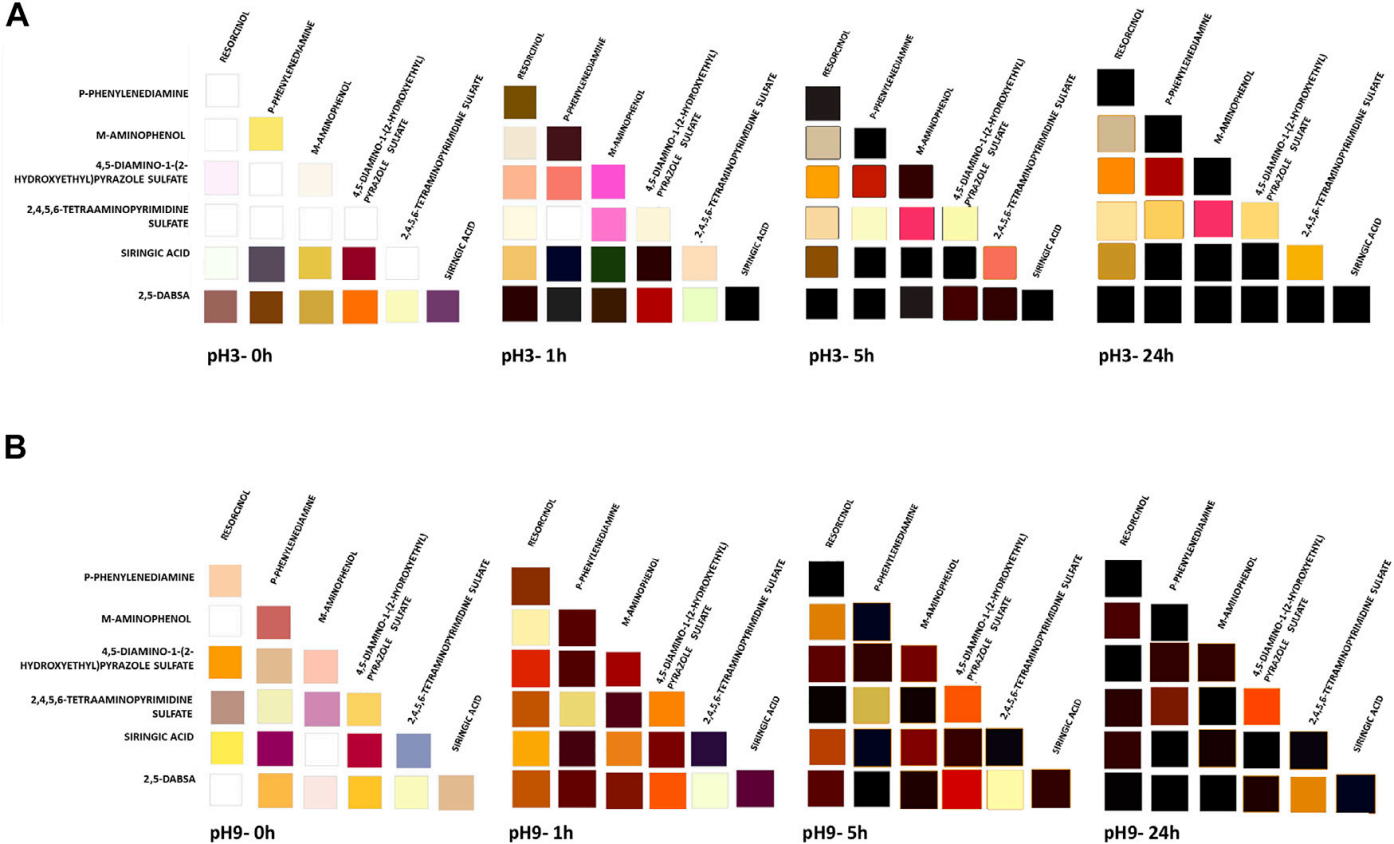
Colors obtained in the enzymatic dyeing at pH 3 **(A)** and pH 9 **(B)** of heteromolecular mixture. The solutions were evaluated at different time of laccase incubation (0, 1, 5, and 24 h). The corresponding CIELAB values are reported in Supplementary Table S2.

In the presence of the enzyme, a significant increase in the reaction rate was observed after 1 h of incubation of all heteromolecular mixtures under both pH conditions (Figure 3). In most of the cases, a significant color variation was also detected immediately after the start of the reaction (time 0).

After 5 h of laccase incubation, all solutions, derived by using the combinatorial approach, became colored. As the reaction continued, many of the colors darkened over time due to the extent of the polymerization degree and the increase in polymer concentration in the solution.

Taking all the results together, the color palette was expanded by using the combinatorial approach with respect to the color achieved by oxidation of the single monomers. All these results were used for wood fiber dyeing with the aim of evaluating the performances of each solution. A different effect is conceivable by applying each one.

### *In situ* Dyeing of Wood Fibers

Once defined all the combination for color synthesis, the use of laccase to catalyze the *in situ* dyeing of wood fibers was investigated for each monomer and for each heteromolecular mixture. To this aim, the reactions were performed in a volume of 50 ml per 1.5 g of dried fiber, sufficient to cover the wood fiber bulk. The enzyme incubation was kept for 24 h, in order to achieve the highest yield of colored products on the fiber. The tests were performed under mild reaction conditions: 22 ± 3°C, 1.33 ± 0.01 atm, without stirring aqueous solution, and without any mordant or penetration agent.

Wood fibers treated with laccases only in the absence of oxidizable precursors led to no visible color change. Treatments comprising precursors in the absence of enzyme resulted in no color change across the fibers. In most of the cases, incubation of wood-based bulk with laccases and precursors resulted in evident *in situ* dyeing of the fibers.

**FIGURE 4:**
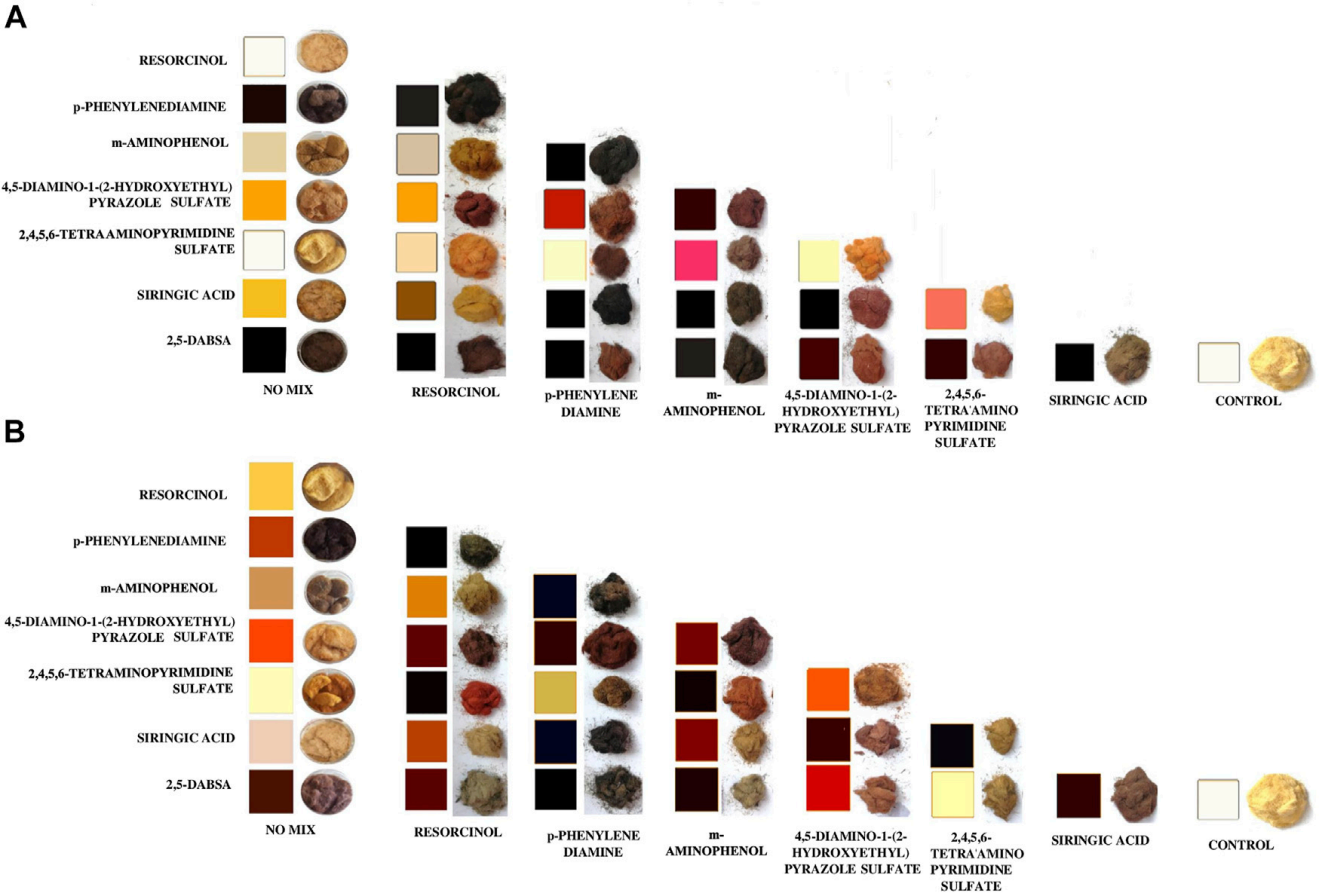
Wood fibers dyed through laccases incubation together with single or coupled monomers in a dyeing bath at pH 3 **(A)** and pH 9 **(B)**. The wood fiber color of each combination was compared with the color related to the corresponding solution.

A diverse range of colors and depths of shade were achieved and homogeneously distributed on the inner and outer parts of the bulk fiber, whereas only few of the coloring solutions were not able to stain the wood fibers. According to the results reported in Figure 4, the reaction products showed remarkable dyeing performances, in terms of intensity of color on the fibers. A variety of orange, red, brown, gray, and black colors was produced. Moreover, one dark green and one dark violet were also synthetized *in situ*. It has also been noticed that some of the dyes generated on the wood fibers totally differed from the color solution related to the corresponding reaction in liquid.

In most of the treated samples, the surface of each wood fiber was homogeneously dyed without uncolored spots. This finding is relevant in comparison with the dyed MDFs that are currently present on the market, whose surface has several uncolored spots on the MDF surface.

### Color Fastness of Water Analysis

Dyed wood fiber samples were subjected to a washing cycle to evaluate the color resistance on this porous material. Undyed or meagerly colored samples were not tested for color fastness. From the data obtained (Table 1), it is possible to detect a slight color reduction of all dyed samples after soaking in hot water. ΔE values were calculated for the colored samples before and after the treatment. ΔE values of all treated samples were less than 7. In particular, the fastness experiment had a relatively noticeable impact on the L* value, along with a minor impact on a* and b* (Supplementary Table S1) (Deng et al., 2009; Zhu et al., 2018).

**TABLE 1:** Color fastness properties of enzymatically dyed wood fibers. The ΔE values were determined by measuring the CIELAB before and after washing treatment (reported in Supplementary Table S1).

Combined precursors	ΔE colors achieved at pH 3	ΔE colors achieved at pH 9
resorcinol; *p*-phenylenediamine	7	5
resorcinol; *m*-aminophenol	1	1
resorcinol; 2,4,5,6-tetraaminopyrimidine sulfate	4	5
resorcinol; 4,5-diamino-1-(2-hydroxyethyl)pyrazole sulfate	7	4
resorcinol; syringic acid	-	-
resorcinol; 2,5-diaminobenzenesulfonic acid	6	-
*p*-phenylenediamine; *m*-aminophenol	3	6
*p*-phenylenediamine; 2,4,5,6-tetraaminopyrimidine sulfate	4	6
*p*-phenylenediamine; syringic acid	1	6
*p*-phenylenediamine; 2,5-diaminobenzenesulfonic acid	6	7
*p*-phenylenediamine; 4,5-diamino-1-(2-hydroxyethyl)pyrazole sulfate	5	6
*m*-aminophenol; syringic acid	6	
*m*-aminophenol; 2,5-diaminobenzenesulfonic acid	7	
4,5-diamino-1-(2-hydroxyethyl)pyrazole sulfate; 2,4,5,6-tetraaminopyrimidine sulfate	5	5
4,5-diamino-1-(2-hydroxyethyl)pyrazole sulfate; 2,5-diaminobenzenesulfonic acid	6	4
4,5-diamino-1-(2-hydroxyethyl)pyrazole sulfate; syringic acid	6	4
2,4,5,6-tetraaminopyrimidine sulfate; syringic acid	-	-
2,4,5,6-tetraaminopyrimidine sulfate; 2,5-diaminobenzenesulfonic acid	5	-
*m*-aminophenol; 4,5-diamino-1-(2-hydroxyethyl)pyrazole sulfate	4	6
*m*-aminophenol; 2,4,5,6-tetraaminopyrimidine sulfate	4	6
syringic acid; 2,5-diaminobenzenesulfonic acid	6	3

The standard deviation was less than 1 unit of ΔE, for each measurement.

### Water Contact Angle on the Wood Fibers

**FIGURE 5:**
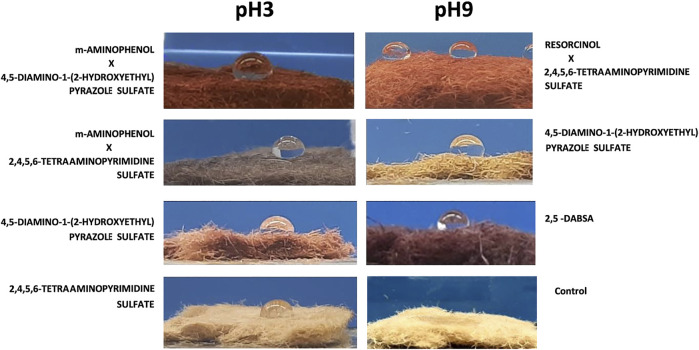
Water drop depositions of the less absorbent wood fibers dyed with laccase and single precursor solutions or heteromolecular mixtures. 20 ul of water solution was dropped on the pressed fiber surface, and the picture was taken after 2 s from the deposition only for the droplet that was not immediately absorbed. The controls were incubated with the same enzymatic solutions at both pH in absence of precursors. They displayed the same behavior and one of them is reported in picture as reference.

The water contact angle was measured with a deionized water droplet on the surface of the form-pressed dried sample at 22 ± 3°C (Table 2). As predictable, water in contact with the dried surface of the form-pressed wood fibers was rapidly absorbed (providing that the contact angle tends to be zero). The 20 µL water was absorbed in <10 s by a consistent number of wood fiber surfaces. However, for eighteen of the samples, the contact angles range from 57° to 92°. The larger the wetting tendency, the smaller will be the contact angle or the surface tension. The time for drop absorption of six samples was less than 1 min, whereas the water drops in contact with the surface of seven of them were still detectable after 5 min (Figure 5). Some of the wood fiber surfaces absorbed water in more than 15 min, in particular those treated with solutions containing *m*-aminophenol, 2,4,5,6-tetraaminopyrimidine sulfate, or 4,5-diamino-1-(2-hydroxyethyl)pyrazole sulfate. In detail, the incubation of wood fibers with laccase and 4,5-diamino-1-(2-hydroxyethyl)pyrazole sulfate at pH 9 allowed the surfaces not to absorb water drops for about 70 min.

**TABLE 2:** Water contact angles (in grade) and drop adsorption time (in minutes) of dyed wood fibers.

pH 3	resorcinol	*p*-phenylenediamine	*m*-aminophenol	4,5-diamino-1-(2-hydroxyethyl)pyrazole sulfate	2,4,5,6-tetraaminopyrimidine sulfate	syringic acid	2,5-diaminobenzenesulfonic acid	Control
resorcinol	92° (0.1 min)	----------	----------	----------	----------	----------	----------	----------
*p*-phenylenediamine	82.5° (1.1 min)	Spreading^a^	----------	----------	----------	----------	----------	----------
*m*-aminophenol	57° (0.1 min)	83° (3.5 min)	Spreading^a^	----------	----------	----------	----------	----------
4,5-diamino-1-(2-hydroxyethyl)pyrazole sulfate	89° (2.3 min)	Spreading^a^	80° (17.0 min)	86° (6.0 min)	----------	----------	----------	----------
2,4,5,6-tetraaminopyrimidine sulfate	Spreading^a^	73° (0.2 min)	88° (19.0 min)	85° (1.0 min)	86° (5.30 min)	----------	----------	----------
syringic acid	Spreading^a^	88° (0.2 min)	106° (1.1 min)	Spreading^a^	Spreading^a^	Spreading^a^	----------	----------
2,5-diaminobenzenesulfonic acid	Spreading^a^	72° (0.2 min)	Spreading^a^	Spreading^a^	Spreading^a^	Spreading^a^	Spreading^a^	----------
----------	----------	----------	----------	----------	----------	----------	----------	Spreading^a^

pH 9	resorcinol	*p*-phenylenediamine	*m*-aminophenol	4,5-diamino-1-(2-hydroxyethyl)pyrazole sulfate	2,4,5,6-tetraaminopyrimidine sulfate	syringic acid	2,5-diaminobenzenesulfonic acid	Control
resorcinol	Spreading^a^	----------	----------	----------	----------	----------	----------	----------
*p*-phenylenediamine	Spreading^a^	Spreading^a^	----------	----------	----------	----------	----------	----------
*m*-aminophenol	Spreading^a^	Spreading^a^	Spreading^a^	----------	----------	----------	----------	----------
4,5-diamino-1-(2-hydroxyethyl)pyrazole sulfate	Spreading^a^	Spreading^a^	82° (0.2 min)	90° (70.0 min)	----------	----------	----------	----------
2,4,5,6-tetraaminopyrimidine sulfate	95° (15.3 min)	Spreading^a^	77° (0.2 min)	Spreading^a^	Spreading^a^	----------	----------	----------
syringic acid	Spreading^a^	Spreading^a^	Spreading^a^	Spreading^a^	Spreading^a^	Spreading^a^	----------	----------
2,5-diaminobenzenesulfonic acid	Spreading^a^	Spreading^a^	Spreading^a^	Spreading^a^	Spreading^a^	Spreading^a^	85° (24.0 min)	----------
----------	----------	----------	----------	----------	----------	----------	----------	Spreading^a^

a Spreading: the water droplet is completely spread out/absorbed on the solid surface.

The standard deviation for each sample was less than 4%.

## Discussion

Laccases can efficiently catalyze polymerization of phenolic and aromatic moieties. Polymerization is initiated by the formation of a radical cation, followed by intermolecular attack to produce dimers (Mikolasch and Schauer, 2009). Colored oligomers and polymers can be synthesized from dimers, miming the natural phenomenon responsible for synthesis of flavonoid polymers. Finally, monomers can be blended into oxidative solutions, thus promoting synthesis of heteropolymers and expanding the color varieties (Jeon et al., 2010; Sousa et al., 2016).

In this work, an innovative and environmentally-friendly process for wood fiber dyeing, based on laccase-catalyzed oxidation, was developed. Seven monomers were selected, from a list of commercially available precursors, for their chemical structures and for their oxidation potential.

Resorcinol is a non-expensive catechol isomer that has already been used as a substrate to produce colored homopolymers and heteropolymers via laccase-catalyzed oxidation and has also been exploited in laccase-catalyzed fiber dyeing (Durairaj, 2005; Sun et al., 2013; Reena et al., 2014; Sýs et al., 2017).

*p*-Phenylenediamine is a monocyclic aryl amine compound, a white to light purple powder, which, when oxidized, turns red first, then brown, and then finally black on exposure to air. It has been used in the past as an ingredient of oxidative hair coloring products or as a textile dye and also as a photographic developing agent (Latha Saranya et al., 2014).

4,5-diamino-1-(2-hydroxyethyl)pyrazole sulfate is a promising candidate as new primary intermediates, considering that it may decrease allergenic risks (Ramadan et al., 2021). As a fact, several patents on diaminopyrazoles and triaminopyrazoles as primary intermediates has been recently granted (Cotteret, 2002; Chassot and Braun, 2003; Ernst et al., 2006; Vidal and Fadli, 2007; Lim et al., 2008; Thuring et al., 2014).

2,5-diaminobenzenesulfonic acid has been already used successfully for the *in situ* coloration of textile fibers, coupled to catechol and resorcinol (Calafell et al., 2007; Pezzella et al., 2016; Zerva et al., 2019). *m*-Aminophenol is already used to increase the number of polymer products because its polymerization mechanism is similar to that of aniline, thus differing from the widely used o-isomer (Dinç et al., 2014).

2,4,5,6-tetraaminopyrimidine, like other tetraaminopyrimidines and triaminopyrimidines, is a heterocyclic dye precursor considered as alternatives to carbocycles in dyes (Morel and Christie, 2011). Renewed interest in pyridine derivatives is indicated by claims as primary intermediates and as couplers in several patents.

Syringic acid is one of the abundant phenolic compounds present in several plants (Srinivasulu et al., 2018), which contributes to the structural integrity of the lignin. Its oxidation in the presence of laccase has been already reported (Jeon et al., 2012). The formation of oligo(phenylene oxide)s to hexamers has been observed, but the detailed characterization of the oligomers has not been well understood. The presence of syringic acid in lignin may act as a good substrate for fungal laccases, a fact that has great importance in the bioremediation and pulp industry (Abe et al., 2005). Furthermore, syringic acid–based synthetic lignin polymers have been prepared using laccase-catalyzed polymerization (Ikeda et al., 1996).

To get insights into heteromolecular mixture contribution to the colored products generated through laccase catalysis, products belonging to homopolymerization and heteropolymerization reactions under acidic and alkaline pH were compared. As it also happens in the case of the hair dyeing process, the reactions are mostly promoted by high pH (Da França et al., 2015), despite POXA1b catalytic properties, whose activity is increased under acidic conditions. The apparent incongruency can be explained by the structure of the active site of laccases and the different nature of non-phenolic substrates such as ABTS, which is generally used as a reference to measure laccase activity, with respect to the dye precursors. The active site of laccases contains four copper ions: a mononuclear copper ion (T1) and a trinuclear copper T2/T3 cluster (Janusz et al., 2020), and the catalysis follows three major steps: T1 copper is reduced by accepting electrons from the reducing substrate, then electrons are transferred from the mononuclear copper to the T2/T3 cluster, and finally molecular oxygen is reduced to water at the T2/T3 cluster.

As far as ABTS is concerned, whose oxidation does not involve protons and has a minimal redox potential, the activity of laccase decreases when pH increases, as a result of the possible involvement of inhibition at the T2/T3 center (Xu, 1997). For a reducing substrate, whose oxidation involves protons, such as phenols, and has a significant redox potential dependence on pH, the pH activity profile of laccase could increase, reflecting the pH-induced redox potential change on both the T1 center and substrate (Xu, 1997).

Solubilization of two different monomers into the same solution results in the increasing rate of color formation. This effect is even more pronounced at pH 3, where the reactivity of the monomers resulted remarkably reduced. A heteromolecular mixture also has the advantage of expanding the chromatic scale of the oxidized solutions. The production of new colors is not due to the presence of more than one homopolymer in the same solution, whose chromatic contribution would overlap, but are related to the formation of new heteropolymeric compounds. In fact, when the single monomer solutions are separately oxidized through laccases and then blended together, the samples display color values that differ from those obtained by oxidizing the monomers together in the same solution. Laccases can promote heteromolecular coupling reactions when two or more substrates are incubated together (Pilz et al., 2003; Jeon et al., 2012). Due to the nature of the products, the heteropolymers have a different absorption profile in comparison with the one with the presence of two homopolymers in the same solution.

**FIGURE 6:**
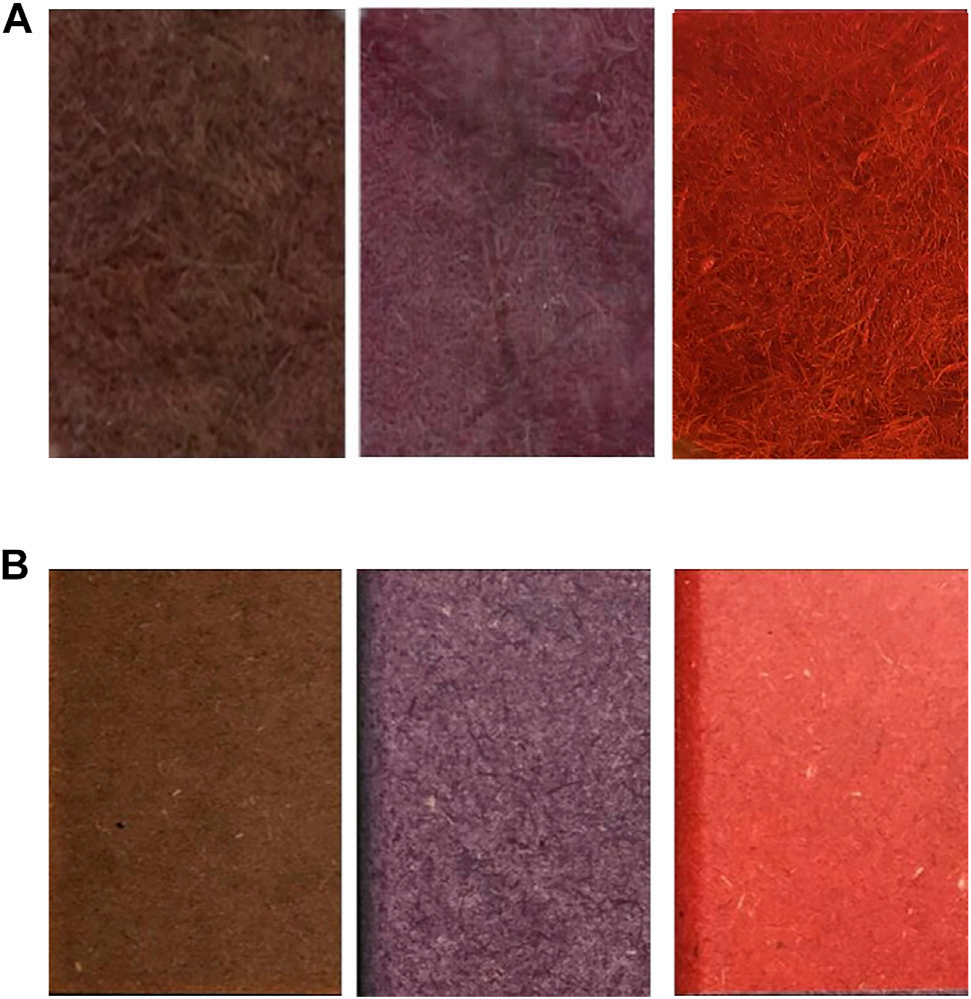
Comparison of dried pressed wood fiber panels that were previously dyed toward laccase catalysis **(A)** with the commercially dyed MDF already present on the market **(B)**.

As previously reported for other fibrous materials (Jeon et al., 2010; Pezzella et al., 2016; Kim et al., 2017; Agrawal et al., 2018; Prajapati et al., 2018), radical-induced polymerizations can take place in the fibers of porous materials and stably dye them through both the steric entrapment of the polymer and the chemical bonding occurring between the colored polymers and the fibers. Despite the absence of stirring and the mild reaction conditions, which allow containing the process costs, the color achieved in most of the reactions is homogeneously distributed over the entire surface of the fibers, even on the innermost part of the batch, keeping its “wooden natural” surface (Figure 6A). Conversely, the stained fibers of commercially available MDFs are impregnated with organic coloring agents and are chemically bound to one another by a special resin and come in different shades (Figure 6B). As reported in the related datasheet, this variation may be seen on the same surface, between the two faces of the same panel, or between the different production batches or different thicknesses. To minimize this effect, the supply must be from a single production. The MDFs fabricated by using wood fibers dyed through laccase could provide a more homogeneous color surface and more reproducible batches with respect to the panels already present on the market. Moreover, the laccase-based dyeing process was found to be more sustainable than the organic-based one, which uses more recalcitrant and less effective dyestuffs.

The performances of the dyeing process, together with its mild conditions, the presence of natural catalyzer, and the absence of recalcitrant chemicals, make the wood fiber dyeing through laccase highly advantageous and ecologically more sustainable. It has been already quoted that some of the dyes generated on the wood fibers display a color significantly different from their dye bath. Since lignin can be a substrate of laccases (Perna et al., 2020), lignin itself, or monomers obtained from its oxidative degradation and can be included in the polymerization reaction; thus, some dyes synthetized *in situ* differ completely from their liquid counterpart. However, the reported difference in color values points out the need to test the conditions of each reaction on wood fibers in order to assess the achievement of a specific color point.

The color generated from the laccase oxidative reaction generally lead to achieving a satisfactory fastness on different materials such as cotton, wool, silk, leather, and nylon (Kim et al., 2007; Sun et al., 2014; Pezzella et al., 2016; Prajapati.et al., 2018; Antunes Barros et al., 2019; Atav et al., 2021). The wood fiber’s color resistance to fading and running confirms a strong binding between the dye and the fiber, with satisfactory color fastness of water property (Zhu et al., 2018) in comparison with previous studies on the fastness of the dyed wood material (Deng et al., 2009). From this point of view, a further advantage of the use of laccases in the dyeing process could be provided by their grafting action on the wood fibers, which could allow the covalent binding of the dye to the ligneous surface (Felby et al., 2002; Schubert et al., 2015; Slagman et al., 2018). Further studies to better explain this phenomenon are still needed.

Some of the laccase-treated wood fibers were less permeable to water (Francisca et al., 2003; Malkov et al., 2004; Iovino et al., 2018). This characteristic shed light to the possibility to prepare an innovative eco-friendly MDF with less impermeabilization agents based on the petrochemical industry.

The improved characteristics of laccase-treated wood fibers over conventional processing methods allow adopting a simpler and milder manufacturing process that limits the use of harsh chemicals and energy consumption and allow producing innovative MDFs three dimensionally dyed with natural textures, addressing the industrial and technical needs required by the market.

## Data Availability

The original contributions presented in the study are included in the article/Supplementary Material; further inquiries can be directed to the corresponding author.
