# Supplementary material for: In Situ Wood Fiber Dyeing Through Laccase Catalysis for Fiberboard Production
Source: Front Bioeng Biotechnol. 2021 Dec 3;9:778971. doi: 10.3389/fbioe.2021.778971 (PMC8678495; doi:10.3389/fbioe.2021.778971)
Supplement: Supplementary file 1 [file Table1.DOCX]

**Table S1 - CIELab of enzymatically dyed wood fibres before and after the washing treatment.** Color values were measured to evaluate the related color fastness properties of the samples (Table 1).

| Molecules | color achieved at pH3 | | | | | | color achieved at pH9 | | | | | |
| --- | --- | --- | --- | --- | --- | --- | --- | --- | --- | --- | --- | --- |
|  | pre- washing | | | post- washing | | | pre- washing | | | post- washing | | |
|  | L* | a* | b* | L* | a* | b* | L* | a* | b* | L* | a* | b* |
| resorcinol; p-phenylenediamine | 18.2 | 3.7 | 8.1 | 25.4 | 3.4 | 8.1 | 18.7 | 3.6 | 8.3 | 24.1 | 3.6 | 8.5 |
| resorcinol; m-aminophenol | 5.0 | 5.0 | 5 | 5.1 | 4.3 | 5.0 | 36.7 | 9.0 | 22.4 | 37.4 | 9.0 | 22.5 |
| resorcinol; 2,4,5,6-tetraaminopyrimidine sulfate | 48.6 | 25.0 | 32.6 | 51.0 | 25.0 | 30.0 | 27.8 | 8.8 | 2.4 | 32.0 | 8.5 | 2.1 |
| resorcinol; 4,5-diamino-1-(2-hydroxyethyl)pyrazole sulfate | 28.2 | 21.7 | 13.5 | 32.8 | 26.0 | 15.5 | 27.8 | 10.0 | 9.8 | 32.1 | 10.1 | 9.9 |
| resorcinol; syringic acid | n.d. | n.d. | n.d. | n.d. | n.d. | n.d. | n.d. | n.d. | n.d. | n.d. | n.d. | n.d. |
| resorcinol; 2,5-diaminobenzenesulfonic acid | 26.0 | 11.8 | 12.6 | 29.4 | 12.0 | 8.0 | n.d. | n.d. | n.d. | n.d. | n.d. | n.d. |
| p-phenylenediamine; m-aminophenol | 10.9 | 4.4 | 8.1 | 13.5 | 4.5 | 8.5 | 21.2 | 7.0 | 8.7 | 25.0 | 11.0 | 8.4 |
| p-phenylenediamine; 2,4,5,6-tetraaminopyrimidine sulfate | 23.4 | 11.7 | 13.8 | 27.0 | 11.5 | 13.6 | 21.2 | 7.0 | 8.7 | 21.6 | 10.0 | 9.0 |
| p-phenylenediamine; syringic acid | 11.3 | 1.6 | 1.7 | 12.0 | 1.6 | 1.6 | 21.5 | 6.8 | 9.8 | 27.3 | 7.2 | 9.6 |
| p-phenylenediamine; 2,5-diaminobenzenesulfonic acid | 28.7 | 11.6 | 16.3 | 34.3 | 13.0 | 18.0 | 26.0 | 6.4 | 11.3 | 32.8 | 6.5 | 11.0 |
| p-phenylenediamine; 4,5-diamino-1-(2-hydroxyethyl)pyrazole sulfate | 30.6 | 13.6 | 13.8 | 36.0 | 13.2 | 13.7 | 14.2 | 15.2 | 12.2 | 19.7 | 15.3 | 11.9 |
| m-aminophenol; syringic acid | 29.6 | 4.5 | 13.5 | 36.0 | 4.3 | 14.2 | n.d. | n.d. | n.d. | n.d. | n.d. | n.d. |
| m-aminophenol; 2,5-diaminobenzenesulfonic acid | 28.2 | 4.4 | 12.9 | 34.8 | 4.3 | 12.5 | n.d. | n.d. | n.d. | n.d. | n.d. | n.d. |
| 4,5-diamino-1-(2-hydroxyethyl)pyrazole sulfate 2,4,5,6-tetraaminopyrimidine sulfate | 59.7 | 21.7 | 44.2 | 64.5 | 21.4 | 44.1 | 33.0 | 14.2 | 24.1 | 38.0 | 14.6 | 24.1 |
| 4,5-diamino-1-(2-hydroxyethyl)pyrazole sulfate; 2,5-diaminobenzenesulfonic acid | 24.3 | 19.2 | 21.0 | 29.9 | 19.2 | 18.0 | 16.4 | 15.2 | 16.2 | 20.2 | 15.5 | 16.2 |
| 4,5-diamino-1-(2-hydroxyethyl)pyrazole sulfate; syringic acid | 37.8 | 22.4 | 17.6 | 43.4 | 22.3 | 17.2 | 38.5 | 17.4 | 16.3 | 42.3 | 17.0 | 16.4 |
| 2,4,5,6-tetraaminopyrimidine sulfate; syringic acid | n.d. | n.d. | n.d. | n.d. | n.d. | n.d. | n.d. | n.d. | n.d. | n.d. | n.d. | n.d. |
| 2,4,5,6-tetraaminopyrimidine sulfate; 2,5-diaminobenzenesulfonic acid | 18.7 | 15.3 | 14.7 | 23.5 | 15.3 | 12.8 | n.d. | n.d. | n.d. | n.d. | n.d. | n.d. |
| m-aminophenol; 4,5-diamino-1-(2-hydroxyethyl)pyrazole sulfate | 12.7 | 10.9 | 10.5 | 17.2 | 10.8 | 10.5 | 15.8 | 10.6 | 7.4 | 21.7 | 10.3 | 7.2 |
| m-aminophenol; 2,4,5,6-tetraaminopyrimidine sulfate | 14.1 | 7.1 | 8.6 | 18.0 | 6.9 | 8.3 | 18.3 | 12.7 | 15.3 | 23.8 | 12.9 | 15.1 |
| syringic acid; 2,5-diaminobenzenesulfonic acid | 24.0 | 8.5 | 12.0 | 29.5 | 8.6 | 9.0 | 10.3 | 11.4 | 9.7 | 13.1 | 11.1 | 9.7 |

n.d.: not determined
