# Supplementary material for: In Situ Wood Fiber Dyeing Through Laccase Catalysis for Fiberboard Production
Source: Front Bioeng Biotechnol. 2021 Dec 3;9:778971. doi: 10.3389/fbioe.2021.778971 (PMC8678495; doi:10.3389/fbioe.2021.778971)
Supplement: Supplementary file 2 [file Table2.DOCX]

**Table S2 - Color value between homopolymer blending at pH3 and pH9.**

Each monomer was separately incubated at three different times and mixed later. Each L*a*b* set of data is related to a blending solution achieved by combining a monomer reported in the row with a monomer reported in the column. The oblique lines separate the values registered at acid condition from the ones achieved in alkaline environment. The standard deviations resulted less than 3 units of ΔE for each measurement.

**1h 5h 24h**

**pH9**

**pH9**

**pH9**

**pH3**

**pH3**

**pH3**

| **resorcinol** |
| --- |
| **p-phenylenediamine** |
| **m-aminophenol** |
| **4,5-diamino-1-(2-hydroxyethyl)pyrazole sulfate** |
| **2,4,5,6-tetraaminopyrimidine sulfate** |
| **syringic acid** |
| **2,5-diaminobenzenesulfonic acid** |

| **resorcinol** | **p-phenylenediamine** | **m-aminophenol** | **4,5-diamino-1-(2-hydroxyethyl)pyrazole sulfate** | **2,4,5,6-tetraaminopyrimidine sulfate** | **syringic acid** | **2,5-diaminobenzenesulfonic acid** | **resorcinol** | **p-phenylenediamine** | **m-aminophenol** | **4,5-diamino-1-(2-hydroxyethyl)pyrazole sulfate** | **2,4,5,6-tetraaminopyrimidine sulfate** | **syringic acid** | **2,5-diaminobenzenesulfonic acid** | **resorcinol** | **p-phenylenediamine** | **m-aminophenol** | **4,5-diamino-1-(2-hydroxyethyl)pyrazole sulfate** | **2,4,5,6-tetraaminopyrimidine sulfate** | **syringic acid** | **2,5-diaminobenzenesulfonic acid** |
| --- | --- | --- | --- | --- | --- | --- | --- | --- | --- | --- | --- | --- | --- | --- | --- | --- | --- | --- | --- | --- |
