# Supplementary material for: In Situ Wood Fiber Dyeing Through Laccase Catalysis for Fiberboard Production
Source: Front Bioeng Biotechnol. 2021 Dec 3;9:778971. doi: 10.3389/fbioe.2021.778971 (PMC8678495; doi:10.3389/fbioe.2021.778971)
Supplement: Supplementary file 3 [file Table3.DOCX]

| **resorcinol** |
| --- |
| **p-phenylenediamine** |
| **m-aminophenol** |
| **4,5-diamino-1-(2-hydroxyethyl)pyrazole sulfate** |
| **2,4,5,6-tetraaminopyrimidine sulfate** |
| **syringic acid** |
| **2,5-diaminobenzenesulfonic acid** |
|  |
|  |

**Table S3 - Color values of dual monomer blending solutions at pH3 (A) and pH9 (B).**

Each L*a*b* set of data is related to a blending solution achieved by combining one monomer reported in the row with a monomer reported in the column. The oblique lines separate the values registered in the presence (+) and in absence (-) of laccases. The values are the means of triplicate samples. The standard deviations resulted less than 3 units of ΔE for each color values measurement.

**0h 1h 5h 24h**

**A**

| **resorcinol** | **p-phenylenediamine** | **m-aminophenol** | **4,5-diamino-1-(2-hydroxyethyl) pyrazole sulfate** | **2,4,5,6-tetraaminopyrimidine sulfate** | **syringic acid** | **2,5-diaminobenzenesulfonic acid** | **resorcinol** | **p-phenylenediamine** | **m-aminophenol** | **4,5-diamino-1-(2-hydroxyethyl)**  **pyrazole sulfate** | **2,4,5,6-tetraaminopyrimidine sulfate** | **syringic acid** | **2,5-diaminobenzenesulfonic acid** | **resorcinol** | **p-phenylenediamine** | **m-aminophenol** | **4,5-diamino-1-(2-hydroxyethyl)**  **pyrazole sulfate** | **2,4,5,6-tetraaminopyrimidine sulfate** | **syringic acid** | **2,5-diaminobenzenesulfonic acid** | **resorcinol** | **p-phenylenediamine** | **m-aminophenol** | **4,5-diamino-1-(2-hydroxyethyl)**  **pyrazole sulfate** | **2,4,5,6-tetraaminopyrimidine sulfate** | **syringic acid** | **2,5-diaminobenzenesulfonic acid** |
| --- | --- | --- | --- | --- | --- | --- | --- | --- | --- | --- | --- | --- | --- | --- | --- | --- | --- | --- | --- | --- | --- | --- | --- | --- | --- | --- | --- |

| **resorcinol** |
| --- |
| **p-phenylenediamine** |
| **m-aminophenol** |
| **4,5-diamino-1-(2-hydroxyethyl)pyrazole sulfate** |
| **2,4,5,6-tetraaminopyrimidine sulfate** |
| **syringic acid** |
| **2,5-diaminobenzenesulfonic acid** |

**B**

**0h 1h 5h 24h**
