# Supplementary material for: In Situ Wood Fiber Dyeing Through Laccase Catalysis for Fiberboard Production
Source: Front Bioeng Biotechnol. 2021 Dec 3;9:778971. doi: 10.3389/fbioe.2021.778971 (PMC8678495; doi:10.3389/fbioe.2021.778971)
Supplement: Supplementary file 4 [file Image1.pdf]

A

B

C

D

E

F

G

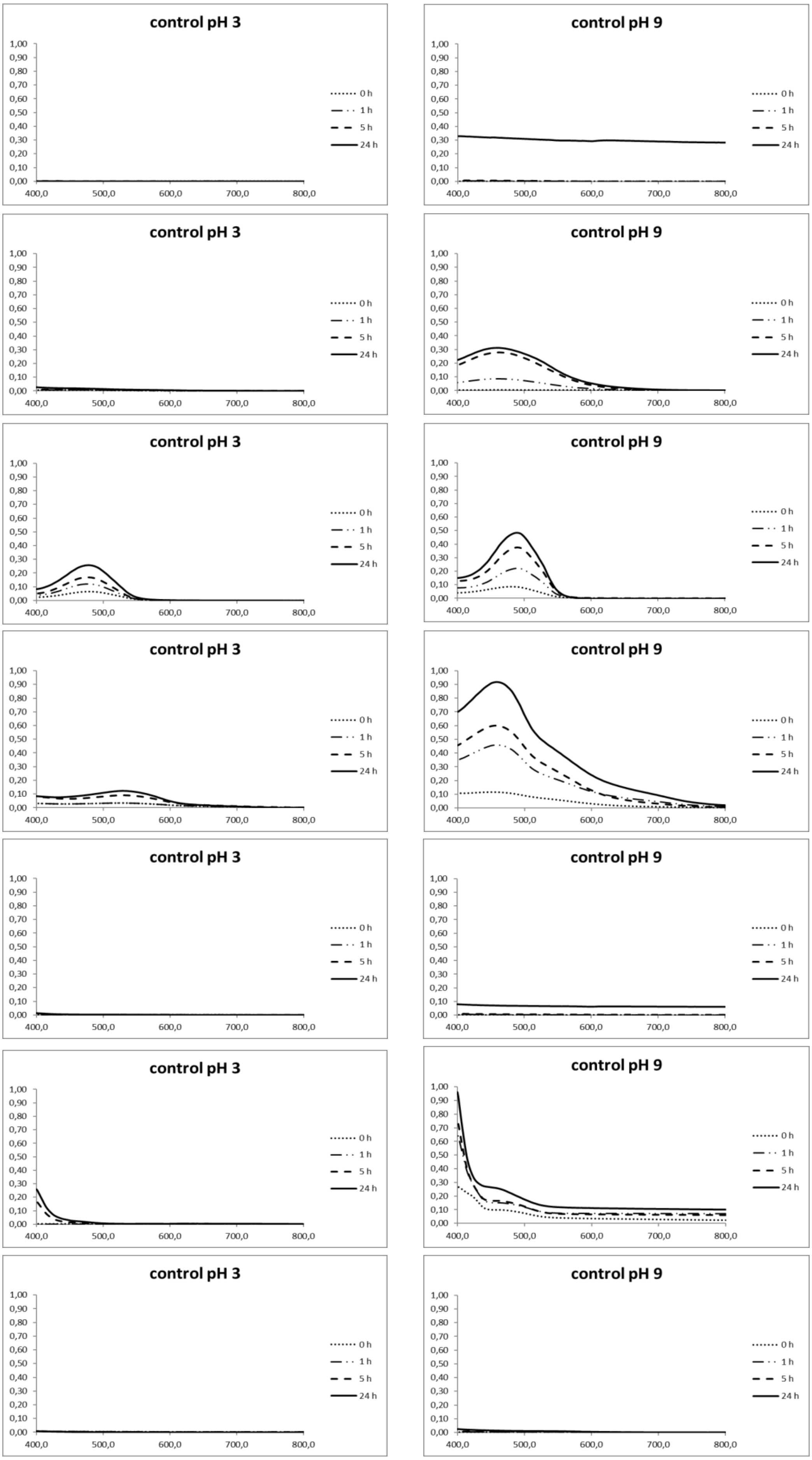

control pH 3
control pH 9
1,00
0,90
0,80
0,70
0,60
0,50
0,40
0,30
0,20
0,10
0,00
400,0
500,0
600,0
700,0
800,0
0 h
1 h
5 h
24 h

Figure S1 –Time-dependent visible light absorption spectra of the control conditions related to the oxidation experiments of each monomer incubated with heat-inactivated laccase solution. Diluted samples incubated with inactivated laccase were indicated in bracket. A – Resorcinol. **B –** p-phenylendiamine. **C –** 4,5-diamino-1-(2-hydroxyethyl)pyrazol sulfate (1h pH9 1:4 diluted; 5h pH9 1:10 diluted; 24h pH9 1:80 diluted). **D –** 2,5-Diaminobenzenesulfonic acid (24h pH9 1:4 diluted). **E –** m-aminophenol. **F -** 2,4,5,6-tetraminopyrimidine sulfate (1h pH9 1:2 diluted; 5h pH9 1:4 diluted; 24h pH9 1:5 diluted). **G –** syringic acid.
